# "I had no idea the university offered"…: The support needs of postgraduate taught students

**DOI:** 10.15694/mep.2021.000121.1

**Published:** 2021-05-11

**Authors:** Amudha Poobalan, John Barrow, Jennifer Cleland

**Affiliations:** 1 University of Aberdeen; 2 Nanyang Technological University Singapore

**Keywords:** Postgraduate taught education (PGT), institutional support, student needs

## Abstract

This article was migrated. The article was marked as recommended.

Introduction

Medical schools have a duty to support students to ensure they fulfil their potential. Relatively little is known about the generic, or therapeutic, support needs of postgraduate taught (PGT) students. This is an important gap to address given the literature suggests that “one size does not fit all”. Thus, our aim was to explore and understand PGT support needs.

Methods

This was a qualitative research study using semi-structured focus groups, conducted in one UK university. We recruited medical and science undergraduates as well as PGT participants to get a sense of what was unique to the PGT student experience. Questions were drawn from the literature and local evaluation data. Discussions were audio-recorded and transcribed verbatim for inductive data coding and analysis.

Results

Six focus groups were conducted with 38 participants. Two main themes each with two sub-themes were identified: Communication (Practicalities; Fulfilled but not tailored) related to the transition into PGT, and Time and Contacts, related to managing the course.

Discussion

PGT students need to address non-academic, often practical, factors in a timely way to navigate university successfully. Knowing who to ask and informal supports are important. Poor and/or difficult-to-access institutional supports may leave PGT students vulnerable.

## Introduction

Postgraduate taught (PGT) courses are a lucrative and burgeoning market for medical schools and related disciplines, many of which offer a suite of face-to-face programmes such as Masters in Medical Sciences, Applied Health Sciences or specialist subjects such as Genomic Medicine, Reproductive biology, Epidemiology and Public Health. Medical schools have a duty, or social contract, to support all students appropriately to ensure they fulfil their potential, but relatively little is known about the support needs of PGT students ( Dhillon, McGowan and Wang, 2008), many of whom are self-funding. This is an important gap to address given the literature suggests that “one size does not fit all” in respect to support, but where institutional-level approaches to enhance student experience ( Guan, Cole and Worthington, 2016) are accessible and well-planned, they promote student success and hence may reduce the need for formal remediation and support linked to poor attainment ( Ciobanua, 2013; Hill, Lomas and MacGregor, 2003: Tinto, 1993; Chou *et al.,* 2019).

Institutional support, often called “therapeutic” support, includes mechanisms that aid integration into university life, such as services for student welfare and pastoral care, financial advice and support, medical and counselling services, and support for student differences such aslearning skills sessions (McInnis, James and Hartley, 2000). While there is a substantial body of research investigating strategies to facilitate the successful transition of first year undergraduate students to university ( Baik, Naylor and Arkoudis, 2015; Thomas 2012; Kebaetse *et al.,* 2018), relatively few studies focus on the transition to postgraduate level, possibly because of an assumption that students who have completed a degree have the necessary skills and knowledge to smoothly move into postgraduate study ( O’Donnell *et al.,* 2009). However, recent studies challenge this assumption ( O’Donnell *et al.,* 2009; Hussey and Smith, 2010; McMillan, 2014; Heussi, 2012; Bunney, 2017). Postgraduate research students describe their transition from degree to postgraduate course as ‘overwhelming’ ( Cluett and Skene, 2006) and ‘difficult’ ( West, 2012) and reporting little institutional support in their journey ( Kinash and Crane, 2016). Studies also suggest that PGT students frequently experience difficulties related to proficiency of language skills, financial constraints, personal safety in an unfamiliar environment, accommodation, culture, prior education and previous exposure to the discipline ( Smith and Khawaja, 2011; Bamber *et al.,* 2019; Gbadamosi, 2018; McPherson, Punch and Graham, 2017).

Providing therapeutic support is not straightforward. The movement towards wider participation in higher education in many countries, including the UK ( Gale and Parker, 2013), and the internationalization of higher education have resulted in a more diverse student population. International students may have obvious cultural and linguistic challenges but the wide variation in the education and socioeconomic backgrounds of home students also has ramifications for successfully managing higher education ( Carpenter, Dearlove and Marland, 2015; Cleland *et al.,* 2013).

Our aim in this paper is to explore and understand PGT student needs and how they use support systems. Our ultimate objective is to provide recommendations to medical schools and related disciplines in respect of how to best support this growing group of students. To get a sense of what was unique to PGT students (if anything) we also collected data from other student groups for comparison.

## Methods

This was a qualitative research study epistemologically grounded in social constructionism ( Crotty, 2003). We used focus group discussions (FGD) to collect multiple perspectives and interpretations of reality and gain a rich understanding of students’ experiences in relation to the research questions ( Morgan and Krueger, 1993).

The study was conducted in the School of Medicine, Medical Sciences and Nutrition at the University of Aberdeen, UK. Institutional support mechanisms are in place to help students with range of issues, such as financial aid and advice, disabilities and specific learning difficulties provision, immigration support, and mental health and wellbeing support. Undergraduate students also have personal tutors to support them throughout their studies.

Using a criterion-based and volunteer sampling strategy ( Patton, 2002) we purposefully targeted students from three specific groups, with the view that these individuals fulfilled our criterion of being able to provide credible information about the topic under study. They were: PGT students; medical and science undergraduate degree students, including a sub-set who entered medicine via a gateway progamme (i.e. widening participation students); and medical students who were intercalating (who had transitioned from medicine into the final year of a science or humanitites degree and who would return to medicine after completing their intercalating year).

We obtained necessary ethical permissions from the University of Aberdeen College of Life Sciences and Medicine Ethics Research Committee. Thereafter, an invitation to take part in the study with a participation information sheet (PIS) was sent to potential participants by email and programme co-ordinators also explained the study at the end of classes. Positive responses were followed up by e-mail, which included further information about the study and focus group discussion (FGD). Focus groups were timed to align with course timetables and held over a three month period (June to November 2017)

Written consent was obtained from each participant prior to conducting the FGDs. At the start of each FGD, the aims of the study were presented and the voluntary nature of participation re-iterated. Reassurances were provided regarding data governance and management. Participants were informed that they were free to withdraw at any point during or after the FGDs without explanation. As all authors had positions of authority in respect to the students, we took care to ensure that each focus group was run by two members of the research team, and at no time did a course coordinator interview his/her student group.

Questions were drawn from the literature on student support and further informed by the findings of previous, internal surveys of the student experience, which included questions on student support. We also explored what institutional student support mechanisms should look like, from the particpants’ perspectives.

FGD were audio-recorded with consent and transcribed verbatim for analysis. Transcripts were managed manually. Data coding and analysis were performed inductively using thematic analysis to generate an initial coding scheme and to look for themes and patterns in the data ( Braun and Clarke, 2006). Preliminary notes and memos were made to record first impressions and further thoughts. Codes were then sorted into categories based on how the different codes were related and linked. After team discussion of preliminary codes and resolution of any coding disagreements, coding and interpretation occurred iteratively and inductively, focusing throughout on the research questions ( Patton, 2002).

## Results/Analysis

Six FGDs representing 339 minutes of data were conducted with 38 participants from medicine, healthcare and science. Two FGDs were conducted with taught postgraduates (PGT 1 and 2: combined n=13: quotes labelled as PGT), two with science undergraduates from first and second year (n=9: labelled as SC) and one each with intercalating medical students (n= 6; fourth years: labelled as IL) and gateway to medicine students (n=10: labelled as G2M). Our focus is PGT data, but data from the other student groups is reported where this is useful for comparison.

Two main themes each with two sub-themes were apparent in the data. Communication (Practicalities; Fulfilled but not tailored) related to the transition into PGT, and Time and Contacts, related to Managing the Course.

### Communication: Practicalities

The processes of registration and acceptance into PGT programmes varied for participants. Some had been accepted onto their course 6-8 months before course commencement, while others were late entrants, with a place confirmed only a few weeks before having to move country/city. However, irrespective of when their place was confirmed, all PGT participants talked about the need for clear, practical information in advance, in respect to the processes of finding accommodation, where teaching would be located (the university is spread over several campuses), timetables, and what steps they needed to go through to get the documents required for day-to-day living (e.g. opening a bank account):

“Earlier communication on timetables in general I think would have been better. Because for me I was working full time and I needed to let them know because I didn’t want to leave my job. But I needed to change my contract, so I needed to know fairly...” (R5 PGT1)

Many participants were not aware of the different campuses, what services were located on each campus, or the distance to their accommodation. For example, student support and advice services were located on the main campus, a 40-minute walk or 20-minute bus journey from the healthcare campus. This led to lot of stress on arrival:

“When I applied for my accommodation, and got it, and then I found it that the accommodation was a bit far from [the heathcare campus].” (R8 PGT1)

“I knew that my campus would be [healthcare] campus. So I thought every information, every facilities, would be going to [healthcare] campus. But after that I had to move to [main campus] to get every information, including the bank account, accommodation details, fee structures, everything.” (R2 PGT1)

Moreover, many participants, irrespective of whether UG or PGT, lacked information about the available student support. They acknowledged that relevant information was included in their welcome communications, but spoke about this not being easily accessible: crucial information was buried in emails and/or the information they received was confusing:

“I think there were links in long emails... I think the information was there but it was all, probably click on this and get to somewhere else and you get from there to somewhere else. You know what I mean. It wasn’t right in front of you.” (R7 PGT1)

### Communication: Fulfilled but not tailored

Students felt they received a lot of generic information in advance, information that was not tailored by course, or to diverse student groups and communities:

“ *I remember getting emails once a month, roughly once a month. I’m presuming from grad school. Very kind of generic emails, saying XXXXXX is a wonderful city and this type of idea. [laughs] Very little detail, about the actual course.” (R1 PGT1)*

“A checklist and things like that might be handy.” (R1 PGT2)

PGTs felt that this advance information was mostly targeted to undergraduates (UGs), citing the example of wondering who their Personal Tutor was only to later learn that only UGs had a personal tutor:

“So from my experience, what I felt was that all this facility has been organised for is undergrad students. Because the meetings you do, the locations, and registration process, everything was made for only...UG.” (R3 PGT1)

“Saying ‘Oh, when do we get allocated our personal tutors?’ I was like ‘That’s [course coordinator] and [course coordinator], you just met them’. And I do think, like I just wrote on the form that, it has a tick box for personal tutors, .....They don’t exist at postgrad.” (R6 PGT1)

PGT students did not necessarily want a Personal Tutor (interestingly the UG data indicated that the usefulness of the Personal Tutor scheme varied widely, associated with the commitment from tutors, as highlighted in previous literature ( Yale *et al.,* 2017)). What vexed them was the confusing communication.

### Managing the course: Contacts

The UG focus group data indicated that this group tended to seek information and advice from their near peers (e.g., students in the year above) and/or administration staff rather than using the University support structures:

“ *she (secretary) put me in contact with the right people. And again, she wrote all the emails. I needed an extension because I was off for a very long time to get a couple of surgeries. And she sorted everything for me, I didn’t have to email anyone asking for an extension. She suggested it. She sorted it out.” (IL2)*

“Yes. From all the different year groups and I guess that could be a kind of structure for support and meeting older students and asking for support, etc. And if you’ve had that initial meeting, you know then you could send someone a message and... I don’t know, like ask for exam help or something or ask for some personal help or something like that.” (R2 SC1)

However, obtaining such informal support was hampered by space and place, particularly for PGT participants. For example, social spaces for students on the healthcare campus were lacking compared to the main campus, and this in turn inhibited opportunities for informal peer support:

“if you did want to go after a class or for a drink or a bite eat with, you didn’t really get that chance to do that because there’s nowhere around here [healthcare campus] to do that.” (R1 PGT2)

“There was a lot more social spaces [at the main campus], I think, to hang out or if you were with other students and stuff.” (IL5)

Once they were aware of support services - usually because they had sought advice about an issue from a peer rather than through official communications - experiences of using services varied:

“But I had no idea that the university offered disability support for things like this.... But again, it’s not something that I would have ever known about if I hadn’t had advice from XXX.” (IL)

“Sometimes when there is a problem, I can’t get an appointment. You could be waiting three, maybe four weeks for one,” (R1 SC2)

### Managing the course: time

A recurring theme in the PGT focus group data was the pressure of time inherent in a 12-month programme. Respondents discussed how there was no time to think, and no “slack” in the system, so any delay (e.g., the release of projects and supervisors, delays in achieving ethical approval) had a knock on impact on other activities:

“It’s just cramped. Everything is happening at the same time”. (R3 PGT2)

“The release of the projects was literally just the week before, wasn’t far before the exams themselves and people never realised, there was an expectation, people had booked holidays to go straight away after exams. When you kind of really need to be around to meet your supervisors and things. We didn’t have time to do it before because the projects had been released late. So there was kind of this whole knock on impact along the...” (R7 PGT2)

## Discussion

Our aim in this qualitative study was to explore PGT student views of therapeutic or institutional support structures and whether these met their needs, and to compare PGT needs and experiences with those of undergraduate students, to elucidate if PGT have specific needs.

Our data indicated that the time constraints of PGT courses are important. The sense of little time to make the transition into PGT and to manage the demands of PGT was palpable in the data. PGT students need timely and tailored pre-enrolment support to help them with practical matters, such as where to live and when to arrive on campus. It is not that undergraduate students do not need or want this support but PGT students do not have as much time as UGs to “find their feet”.

After transitioning into postgraduate study, PGT students become very anxious about delays and roadblocks, perceiving these as impacting adversely on their ability to manage everything in the time available. Given this, we suggest that PGT pre-enrolment and induction materials should focus on practical advice and guidance, as well as highlighting what structures are in place given the perceived availability of support predicts the use of student support services ( Julal, 2015). Second, PGT timings and scheduling need to be planned carefully, to support students with timely task management.

Our respondents struggled to know what formal support structures were available, and how to access these supports. Although they were aware that information had been provided by the institution, most students tended to muddle through, depending on serendipity and peers for information. Informal support seemed at least as important as formal support ( Shadowna, Williamson and Guerrac, 2019). However, PGT students have limited opportunities to develop ties ( Granovetter, 1973) or bridging networks (Putman, 2000) with other groups, unlike undergraduates who are in situ for several years and have the benefit of time to learn the systems and make connections to achieve their goals, such as gathering information ( Croll, 2004). For example, PGT students move on after their course, leaving no “senior” students from whom new PGT students can make links. PGT also have intensive timetables, and less time to engage with opportunities which would facilitate informal support and the acquisition of necessary information. Given this, near-peer mentoring schemes or advice channels (e.g. Frequently asked questions (FAQs) and podcasts prepared by the previous PGT cohort, or alumni support using virtual platforms) may be of value to PGT students, as would the provision of spaces (social and learning) which promote peer interaction and hence student engagement ( Harrop and Turpin, 2013; Waldock *et al.,* 2017). We suggest that the student voice is critical in developing learning and social spaces, as well as therapeutic support structures and how these are communicated.

This study has strengths and limitations. A qualitative approach allowed us to explore student experiences and perceptions of therapeutic support, and our different participant groups allowed a glimpse into the different support needs and access issues for a range of UG and PGT students, rather than focusing on PGT only. The study was conducted in one university so the results may not be transferable to other contexts. The number of participants was relatively small but they had high information power given the narrow research questions, sample specificity and the quality of the data ( Malterud, Siersma and Guassora, 2016). Our participants were self-selecting and arguably represent those who engaged most with the research topic. We interviewed students in their subject groups, so discussion within groups was not dominated by any one group of students. The PGT FGDs were the most “lively”, possibly because of group diversity in terms of background and prior experiences (e.g. Zaitseva and Milsom, 2015).

Qualitative research and analysis are dependent on the relationship between researcher and research process (e.g. Alvesson and Skoldberg, 2000). We considered our positions and relationships with the participants and the data constantly and critically bearing in mind our different disciplinary backgrounds, research interests and personal life courses, and how these might have shaped our co-construction of the data. We minimized contact between authors and their own students, with the view that participants may not have felt comfortable to be open and discuss matters with someone they perceived as a ‘teacher’ or ‘course coordinator’.

## Conclusion

In conclusion, we identified that while PGT students have similar support needs to undergraduate students, lack of time and contacts are obstacles to accessing information and support services. Clear, timely communication and addressing obstacles are needed to help this group manage the transtion into PGT, adjust into postgraduate study and fulfill their potential, while minimising the need for failure-remediaton support.

## Take Home Messages

• Universities typically provide a range of information and support services to help students make the transition into and through their courses.
• Little is known about how postgraduate taught (PGT) students access and use such therapeutic, institutional-level support.
• Nonacademic, often practical, factors may need addressed before students can focus on academic matters. The inherent time limits of PGT courses add pressure.
• The study design did not allow us to identify how PGT manage difficulties and barriers.
• We urge medical schools and universities to focus efforts on developing robust institutional-level support and communication systems which minimise the need for failure-remediation approaches to support.

## Notes On Contributors

Amudha Poobalan MBBS, MSc, PhD (Public Healh), SFHEA, is a Senior Lecturer in Public Health. She has a medical background and has extensive experience in teaching and learning. Her pedagogical interests are improving student experience and strengthening research through improving research skills of health care professionals. ORCID: https://orcid.org/0000-0002-6975-3874

John Barrow BSc(Hons), PhD, PgCert (Higher Education), SFHEA, is a Dean for Entrepreneurship & Employability as well as a Senior Lecturer in Biochemistry & Molecular Biology. He is involved in embedding employability skills into curricula and developing award-winning visual approaches in his life science teaching.

Jennifer Cleland BSc(Hons), MSc, PhD, D Clinical Psychology, FRCP (Edin), FAoME, is Professor of Medical Education Research and Vice-Dean Education, LKC Medicine, Nanyang Technological University Singapore. She publishes extensively in the field of medical education. ORCID: https://orcid.org/0000-0003-1433-9323
