# Supplementary material for: "I had no idea the university offered"…: The support needs of postgraduate taught students
Source: MedEdPublish (2016). 2021 May 11;10:121. [Version 1] doi: 10.15694/mep.2021.000121.1 (PMC10939527; doi:10.15694/mep.2021.000121.1)
Supplement: Peer review history

# Reviewer report: Reviewer response for version 1

**DOI:** 10.21956/mep.18946.r27109

**Published:** 2021-07-16

**Authors:** Richard Hays

**Affiliations:** 1 James Cook University

This review has been migrated. The reviewer awarded 4 stars out of 5 I would like to thank the authors of this article for addressing a not so obvious gap in understanding how best to support PG students. I have always supported investment in student support services, but became aware that PG students accessed these services infrequently. The challenges faced by PG students are different and their sometimes limited campus attendance limits access to available services. Even though most PG students are highly motivated and often have a clearer vision of where they are headed, some do face serious challenges fitting in personal and student life. This research confirms the need for considering different kinds of support services, contributing to a more evidence-based approach. A mild disappointment is that the research seems to have focused on full-time PG students, when part-time PG study is becoming more common and I think that there may be differences. Overall a useful read for leaders of programs and student support services.

Reviewer Expertise:

NA

No decision status is available

# Reviewer report: Reviewer response for version 1

**DOI:** 10.21956/mep.18946.r27108

**Published:** 2021-05-12

**Authors:** Mary Higgins

**Affiliations:** 1 University College Dublin, National Maternity Hospital

This review has been migrated. The reviewer awarded 3 stars out of 5 I want to thank you most sincerely for this paper which I found really interesting. My comments are meant to further improve any future research you may do in this area. -Have you read the COREQ or SRQR criteria for reporting qualitative research? -In particular, did you reflect on how your own perceptions may have shaped your interpretations of the work?

Reviewer Expertise:

NA

No decision status is available
